# Selective Hole Filling of Red Blood Cells for Improved Marker-Controlled Watershed Segmentation

**DOI:** 10.1155/2021/5678117

**Published:** 2021-12-06

**Authors:** Fatih Veysel Nurçin, Elbrus Imanov

**Affiliations:** ^1^Biomedical Engineering, Near East University, 99138 Nicosia TRNC Mersin 10, Turkey; ^2^Computer Engineering, Near East University, 99138 Nicosia TRNC Mersin 10, Turkey

## Abstract

Manual counting and evaluation of red blood cells with the presence of malaria parasites is a tiresome, time-consuming process that can be altered by environmental conditions and human error. Many algorithms were presented to segment red blood cells for subsequent parasitemia evaluation by machine learning algorithms. However, the segmentation of overlapping red blood cells always has been a challenge. Marker-controlled watershed segmentation is one of the methods that was implemented to separate overlapping red blood cells. However, a high number of overlapped red blood cells were still an issue. We propose a novel approach to improve the segmentation efficiency of marker-controlled watershed segmentation. Local minimum histogram background segmentation with a selective hole filling algorithm was introduced to improve segmentation efficiency of marker-controlled watershed segmentation on a high number of overlapping red blood cells. The local minimum was selected on the smoothed histogram for background segmentation. The combination of selective filling, convex hull, and Hough circle detection algorithms was utilized for the intact segmentation of red blood cells. The markers were computed from the resulted mask, and finally, marker-controlled watershed segmentation was applied to separate overlapping red blood cells. As a result, the proposed algorithm achieved higher background segmentation accuracy compared to popular background segmentation algorithms, and the inclusion of corner details improved watershed segmentation efficiency.

## 1. Introduction

According to the World Malaria Report 2018, 219 million cases were reported in 2017 worldwide. Global Health Estimates 2016 stated that malaria is in the top 10 causes of deaths in low-income countries [1]. African region carries the largest burden with 200 million reported cases that make up 92% of all the reports. South-East Asia region comes second with 5%, followed by the Eastern Mediterranean Region with 2% of all the cases [1]. There are 5 species of malaria parasites such as Plasmodium falciparum, Plasmodium vivax, Plasmodium malariae, Plasmodium ovale, and plasmodium knowlesi [2]. In World Malaria Report 2018, Plasmodium falciparum accounted for 99.7% of all the cases in Africa. Malaria diagnosis is carried out manually through a microscopic examination of blood by trained microscopists. The role of the miscroscopist is to determine whether red blood cells (RBCs) are affected by malaria parasites. Once parasitemia is detected, manual counting of parasites is carried out along. Besides detection, counting the number of parasites is required to test drug-resistance and effectiveness and to classify disease progression [2, 3]. However, this manual procedure depends on the skill and experience of the miscroscopist in a strong way. Additionally, there could be external conditions that can also alter the performance of the miscroscopist such as lack of resources to work in isolation. This can lead to an incorrect diagnosis that will end up with false treatment. In false-positive cases, the patient might suffer from side effects of antimalarial drugs whereas in false-negative cases, a misdiagnosis entails unnecessary use of antibiotics and the disease progression into a severe state [2–4]. The lack of reliability of the manual approach encouraged automated diagnosis of malaria with the purpose of providing a standardized diagnosis, in addition, to reducing the workload of field workers and diagnostic cost [3].

A common approach for automated quantification of parasitemia includes a number of steps. The first step is image acquisition, followed by preprocessing to equalize uneven illumination and colour differences between images due to staining conditions. The second step involves the segmentation of individual blood cells in cell clumps for accurate counting of parasites. This step is usually accompanied by postprocessing in order to improve segmentation results by eliminating remaining wastes. Computation of features to describe healthy and nonhealthy (infected) cells in the third step depends on the second step, where segmentation of individual red blood cells in cell clumps affects the correct evaluation of parasites. The number of infected cells versus the total cell ratio totally depends on the correct segmentation of individual red blood cells in cell clumps. The final step involves automated classification. Here, an algorithm should differentiate healthy and infected cells for given images [3, 4].

Uneven illumination and noise are generally handled in the preprocessing step. Grayscale conversion is used widely for illumination correction, and morphological operators are used to remove unwanted noises that are generally smaller than red blood cells [4].

Numerous techniques have been applied to segment red blood cells from their background in order to eliminate unnecessary information presented in the image. Many algorithms were tested on thick and thin blood smears. Global thresholding, adaptive thresholding, histogram-based thresholding, mathematical morphology, and *k*-means clustering methods were used in many studies in order to segment foreground from background. Savkare and Narrote applied global thresholding (Otsu's threshold) on the enhanced green channel to separate red blood cells from the background [5]. An adaptive thresholding technique, followed by mathematical morphological operators, was utilized in a study for noise variations [6]. Mushabe et al. used histogram-based thresholding to extract red blood cells on a limited variety of images [7]. Tek et al. utilized top-hat filtering as an example of morphological segmentation which uses the morphological area. The separation of overlapping red blood cells is an important step in order to accurately count infected red blood cells [8]. Zafari et al. created a novel algorithm to separate partially overlapping objects in silhouette images by an ellipse fitting approach [9]. However, the application of this technique to RBCs failed to give comparable results due to varying shapes of RBCs. A morphological approach for separation of the overlapping RBC was applied by Di Ruberto et al. A hemispherical disk-shaped structuring element was used to enhance the integrity and circularity of the RBCs cells, and a disk-shaped flat structuring element was used to separate overlapping cells in an attempt to improve the efficiency of the watershed algorithm [10]. Savkare and Narote utilized a *k*-means algorithm with a *k* value of 2, to separate background-foreground [11]. This was followed by edge detection and later by watershed transform to separate overlapping cells. Marker-controlled watershed transform is a highly proposed algorithm through biomedical domains and is also introduced to improve the segmentation of overlapping red blood cells. Background markers are pixels that are not part of any object-of-interest and are usually defined as a mask. Foreground markers are connected blobs of pixels inside of each object-of-interest. Each foreground marker is associated with a specific watershed region. To summarize, the number and location of foreground markers contribute to the performance of watershed segmentation. In a study, Radon transform was employed to improve the selection of markers [12]. However, they only succeeded to improve efficiency on running time.

The purpose of this study is to improve individual red blood cell segmentation on malaria blood smear images by watershed segmentation. In that regard, improvements in foreground and background markers were studied.

The proposed work introduces a novel background segmentation algorithm that includes area-limited morphological hole filling. Morphological hole filling is an important step to remove irrelevant data inside the red blood cells. However, this method can be disadvantageous when considering a high number of overlapped red blood cells, where the outer boundaries of a high number of overlapped red blood cells are not informative for marker-controlled watershed segmentation. For that purpose, the hole filling was limited to 1 to 3 RBC sizes. In that way, the inner corners of a high number of overlapped red blood cells (more than 3) were conserved, which was observed to improve watershed segmentation efficiency. The algorithm was tested on samples taken from the Mamic image database [13]. Notable results will be compared in Results and Discussion.

## 2. Materials and Methods

**Figure 1:**
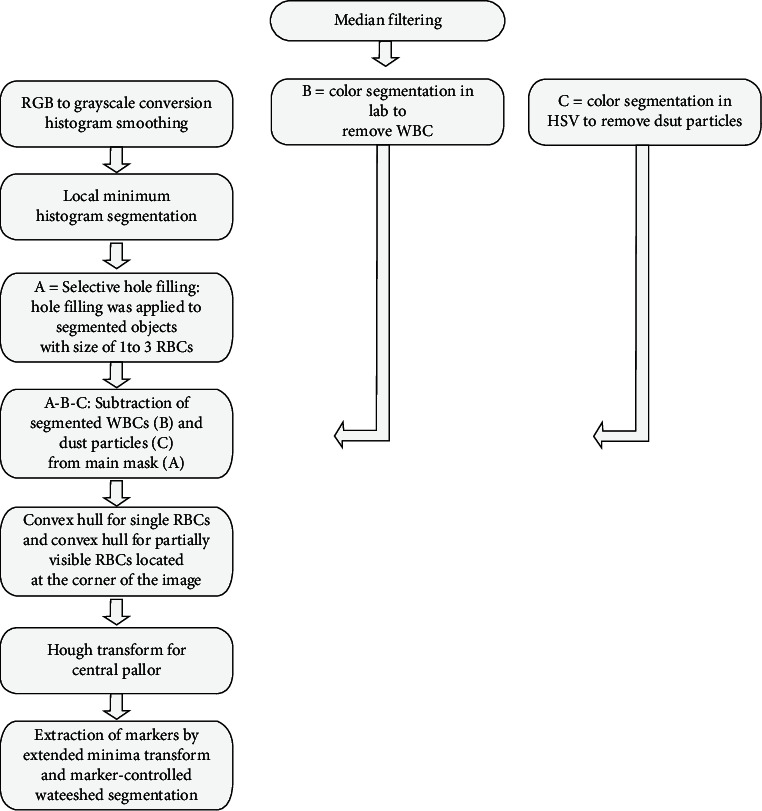
Steps of the algorithm as RBC segmentation (a), WBC segmentation (b), and dust particle segmentation (c).

Malaria blood smear images used in this study were taken from a publicly accessible Mamic image database [13]. Blood smear images are 100x magnified and consist of healthy RBCs, infected RBCs, white blood cells (WBCs), thrombocytes, dust particles, and artifacts. The algorithm was modelled on 66 images that were randomly picked from the dataset. The proposed background segmentation was evaluated on these images. However, the evaluation of the algorithm for overlapping red blood cells requires samples with an abundance of overlapping RBCs. To this end, another set of 15 images was also used to evaluate the algorithm. The proposed algorithm was performed on Matlab 2019a platform. The steps of the algorithm start with the preprocessing of RGB images with median filtering. The median filtered image will be followed by red blood cell segmentation, white blood cell segmentation, and dust particle segmentation sections separately. For the red blood cell segmentation section, more preprocessing methods were followed as RGB to grayscale conversion and histogram smoothing. The second step is the segmentation of red blood cells by the proposed local minimum histogram background segmentation along with Lab colour segmentation of White blood cells and HSV colour segmentation of dust particles. White blood cells and dust particles were segmented to be later removed in the image. The third step is the beginning of postprocessing of the segmented red blood cells. The area filtering by connected components with the purpose of limiting hole filling, named as “selective hole filling,” was applied. After that, morphological operators and selective hole filling (refilling) were followed to remove the remaining wastes. Moreover, the convex hull was applied to both partly visible RBCs positioned at the corner of the image and single RBCs. Additionally, the Hough transform for circle detection was employed to fill bright circular areas at the center of some red blood cells that are known as central pallor. At the final step, marker extraction by the extended minima transform followed by marker-controlled watershed segmentation was applied to separate overlapping RBCs. Following segmentation, pixel connectivity was used to label and count segmented red blood cells in an automated way. These steps are illustrated in Figure 1.

### 2.1. Preprocessing

To preserve the edges of red blood cells, median filtering was applied to the red, green, and blue input image channels. The colour variations between samples due to staining conditions are equalized with the following use of grayscale transformation. Histogram smoothing before local minimum histogram segmentation was used to prevent the selection of false peaks. On the other hand, preprocessing steps for segmentation of WBCs and dust particles only consist of median filtering of RGB channels.

### 2.2. Red Blood Cell Segmentation and Selective Hole Filling

**Figure 2:**
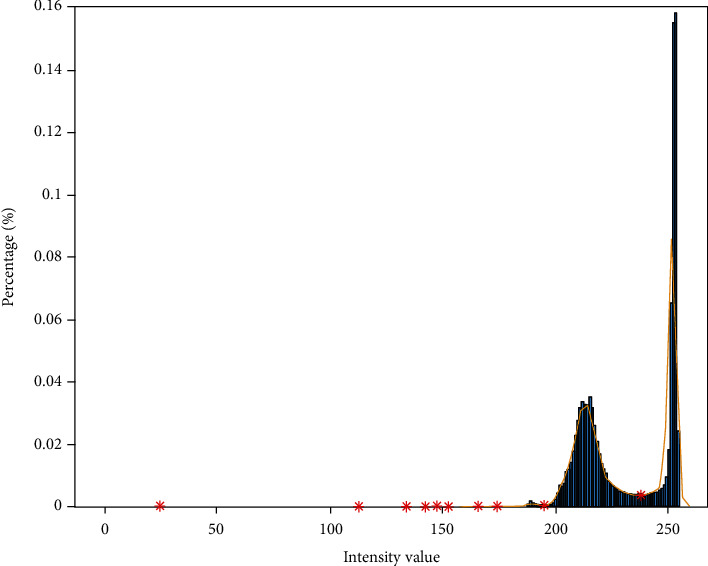
Orange sloping line on the histogram is smoothed histogram, and red stars are detected local minima on smoothed histogram.

The segmentation of red blood cells from the background is the first step of segmentation. That is also defined as background subtraction which plays an important role in the intact extraction of RBCs and removal of irrelevant data. Two groups of pixels were observed in the histogram, with one group representing RBCs and the other group representing the background. In the smoothed histogram, the algorithm seeks the local minimum between the two highest peaks that can be seen in Figure 2. The steps of local minimum detection are explained as follows:

- The highest two peaks in the histogram were detected
- The first peak value (height of the peak) (-0.001) was chosen as the threshold to find peaks in the next step of the algorithm
- All peaks were detected with the threshold that yields the highest two peaks
- Following the first peak, the local minimum (intensity value) was detected. For multiple local minima, the first local minimum was selected for the thresholding point of the histogram

Within the range of the highest two peaks, several local minima can exist. The local minimum detection was designed explicitly in this way as explained to avoid false local minimum detection. The selection of the first local minimum between the highest two peaks contributes to intact red blood cell segmentation. The presence of many local minima can be seen in Figure 2. The intensity value that corresponds to the detected local minimum was chosen as the threshold level to segment red blood cells where the image was converted into a binary mask. Small irrelevant objects were eliminated by area filtering by connected components. Irrelevant objects and residual pixels that are not related to segmented objects were filtered in this way. This approach requires area information of segmented objects in binary masks. Matlab image region analyser app was used to analyse segmented objects. It was determined that objects with an area of less than 750 pixels are not RBCs. As a result, this value was used to remove small irrelevant objects.

After that, segmented objects with 1 to 3 RBCs areas were selectively hole-filled. The morphological hole filling was limited to 3 red blood cell sizes where watershed transformation is suitable for the separation of RBCs in cell that clumps up to this number.

This part of the algorithm plays important role in marker-controlled watershed segmentation, where it is difficult to separate the high number of overlapping red blood cells (more than 3) without corner details of RBCs in cell clumps. These corner details help extraction of foreground markers that contributes to watershed segmentation. Therefore, these inner corner details were preserved for the high number of overlapped red blood cells as morphological hole filling was not applied for these.

Consequently, objects with an area of 1 to 3 RBC sizes were extracted with area filtering by connected components where morphological hole filling was applied to these RBCs. The selective hole-filled mask and postprocessed local minimum histogram segmented mask were combined together with union set operation. The average triple-overlap area was calculated to be 9680 pixels. By adding 10 percent to the average area, the up limit was defined and was approximated to 10750 pixels. The down limit was set as 1000 pixels by 55% of the smallest individual red blood cell region in order to also include partially visible red blood cells. Some overlapping red blood cells that touch the corner of the image could be lost with border cleaning operation. Therefore, border cleaning operation was not used to count as many red blood cells.

### 2.3. Colour Segmentation

White blood cells appear to be intense purple and dust particles vary from light brown to black. Therefore, colour segmentation was employed to remove white blood cells and dust particles as they present distinguishing colours compared to RBCs.

#### 2.3.1. White Blood Cell Segmentation

Hue Saturation Value (HSV) colour segmentation was used in the literature for the segmentation of WBCs [14]. The segmentation was achieved with the manipulation of the hue channel. The HSV colour segmentation can be useful with an image consisting of red blood cells along with white blood cells. The white blood cell can be segmented from the background depending on the distribution of pixels. However, the images in our data rarely have white blood cells where their colour varies due to staining conditions. Therefore, the distribution of pixels cannot be relied on for the segmentation of white blood cells in our dataset. Consequently, colour dimensions were found to be more useful in our dataset. Images were converted to lab colour space to utilize colour dimensions. Later, a and b channels were utilized for white blood cell segmentation where channel “a” refers to red/green and channel “b” refers to blue/yellow value. After the segmentation of the white blood cell, the complement of the segmentation mask was taken that was followed by area filtering to remove residual pixels (objects with an area less than 200 pixels) in the white blood cell boundaries. The complement of the resulted mask was taken, and morphological closing with a disc-shaped structuring element that has a radius of 15 was applied to smooth the edges of the segmented WBC. The morphological dilation with a disc-shaped structuring element that has a radius of 7 was followed to ensure complete segmentation of WBC.

At the final part of white blood cell segmentation step, area filtering was utilized to remove any objects other than white blood cells. The diameter of white blood cells can be small as 10 *μ*m while red blood cells are around 7 *μ*m. In our data, the largest RBCs have an area of around 4000 pixels. Therefore, area filtering with 5000 pixels lower limit would only yield white blood cells while filtering any other object in the image share similar colour dimensions with WBCs such as platelets and malaria parasites. The segmented white blood cell was later subtracted from the red blood cell segmentation mask. As a result, the white blood cell was removed from the image.

#### 2.3.2. Dust Particle Segmentation

Dust particles could contaminate blood smear images. Either in the blood film or on the microscope camera or lens, these dust particles may be present. These dust particles vary in colour from light brown to dark. For their segmentation, the hue channel of the HSV colour space can be used. On the hue channel, the range of brown colour was set to allow dust particles to be segmented. In order to smoothen it, the edge of dust particle morphological image closing with a disc-shaped structuring element that has a radius of 15 was utilized and area filtering was followed to filter very small objects. The small size and irrelevant objects (less than 200 pixels) that were falsely segmented as dust particles were removed by area filtering. Finally, segmented dust particles were subtracted from the red blood cell segmentation mask that results in the removal of dust particles.

### 2.4. Refilling of Holes

The selective hole filling approach that was explained in the preprocessing part was repeated with the same area parameters. In order to prevent the fusion of neighboring RBCs and platelets in the convex hull operation, morphological image opening with disk-shaped structuring elements with a radius of 5 was used before the convex hull step.

### 2.5. Convex Hull

In case of physical damage, some RBCs were disturbed and partially separated. These separate RBCs were encapsulated using the convex hull technique. However, we have limited this method to single-sized RBCs as the application of it to overlapped RBCs can cause loss of RBC boundary details. In other studies, this method was utilized for the detection of single or two overlapped cells [15, 16]. In our study, partially separated single-sized RBCs were extracted by area filtering and the convex hull was used to encapsulate these RCBs for intact segmentation. The convex hull was also utilized for partially visible red blood cells at the corner of the image where RBCs with the small area were extracted and encapsulated by the convex hull. The result of these two convex hull and selective hole refilling masks were united with the union set operation. Final area filtering was applied to ensure the removal of irrelevant objects (less than 750 pixels). The convex hull is defined as follows:

(1) $$\begin{array}{c} \overset{\;|\;x\;|\;}{\underset{i=1}{\sum }}\alpha_{i}x_{i}\;|\;\left(\forall_{u}:\alpha_{i}\geq 0\right),\\ \overset{\left|x\right|}{\underset{i=1}{\sum }}\alpha_{i}=1, \end{array}$$

where ∣*x*∣ defines set of finite points, as *x*_*i*_ is a point in ∣*x*∣. The weight of *x*_*i*_ is *α*_*i*_ while the sum of all normalized weight mean must be equal to 1.

### 2.6. Hough Transform for Circle Detection

In the proposed approach, hole filling of the high number of overlapping red blood cells was avoided which makes the existence of central pallor a problem. The central pallor is segmented as background as it has the same colour intensity as the background. This can cause oversegmentation issues in watershed transformation. In order to overcome this problem, these central pallors were segmented by Hough circle transform with a range of 5 to 25 radius and 88% sensitivity. The segmented circles were dilated with a disk-shaped structuring element that has a radius of 5 to ensure total segmentation of central pallors. These segmented central pallors were united with the previous mask. And finally, the segmentation of red blood cells was completed.

### 2.7. Watershed Segmentation

**Figure 3:**
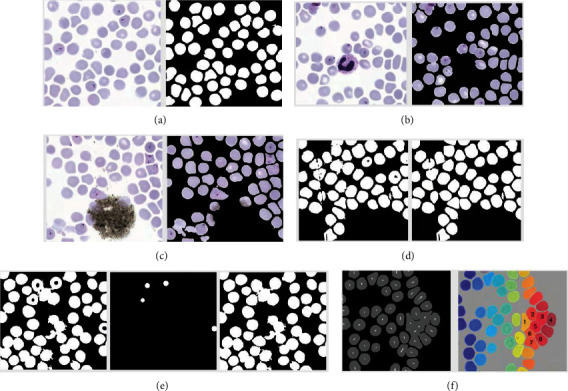
The visual outputs of the algorithm steps. The local minimum histogram background segmentation applied to the input image (a), followed by white blood cell segmentation (b), dust segmentation (c), postprocessing with convex hull (d), and Hough circle detection (e), leading to marker extraction and watershed segmentation (f).

Foreground markers were extracted by distance transformation followed by extended minima transform. After that, watershed segmentation was applied to separate overlapping red blood cells. The segmented red blood cells were covered by a bounding box. The bounding boxes with areas under 500 pixels have not been counted as RBCs. The steps of the algorithm are visually illustrated in Figure 3. Note that each image has different inputs to emphasize the significance of each step. Red blood cell segmentation is illustrated in Figure 3(a), whereas the removal of white blood cell and dust particles is illustrated in Figures 3(b) and Figure 3(c), respectively.  Figure 3(d) shows convex hull application on physically disturbed red blood cells. This helps physically disturbed RBCs to be segmented as one piece instead of being falsely oversegmented. Moreover, Figure 3(e) shows the existence of central pallor for 5 overlapping RBCs. Here, in order to fill those 3 holes (central pallors) to prevent oversegmentation in the next step, Hough transform was employed. Lastly, Figure 3(f), extraction of markers and successful separation of overlapping 8 red blood cells are shown.

### 2.8. Evaluation Method

Assessment of red blood cell segmentation and counting is taken into consideration in this section. To measure the performance of our algorithm, necessary methodologies were acquired. The pixel segmentation accuracy was measured by the Jaccard similarity coefficient, which is a widely used segmentation evaluation technique [17]. The similarity of final segmentation mask A with ground truth mask B was computed with intersection over union. The Jaccard similarity coefficient is expressed as follows:

(2) $$\begin{array}{c} \text{Jaccard}\left(A,B\right)=\frac{\;|\;A\cap B\;|\;}{\;|\;A\cup B\;|\;}, \end{array}$$

where *A* is the final segmentation mask and *B* is the ground truth mask.

For the separation of overlapping red blood cells, the watershed algorithm was used, resulting in a successful counting of red blood cells. RBCs were counted by the connectivity of pixels as cells were separated. The relationship between manually and automatically counted RBCs can be quantified by accuracy, accuracy, recall, and F1 score. For calculating these parameters, the elements of the confusion matrix were used as follows:

(3) $$\begin{array}{c} \text{Accuracy}=\frac{\;|\;\text{TP}+\text{TN}\;|\;}{\;|\;\text{TP}+\text{FP}+\text{TN}+\text{FN}\;|\;}, \end{array}$$

(4) $$\begin{array}{c} \text{Precision}=\frac{\left|\text{TP}\right|}{\;|\;\text{TP}+\text{FP}\;|\;}, \end{array}$$

(5) $$\begin{array}{c} \text{Recall}=\frac{\left|\text{TP}\right|}{\left|\text{TP}+\text{FN}\right|}, \end{array}$$

(6) $$\begin{array}{c} \text{F}1=2\times \frac{\text{precision}\times \text{recall}}{\text{precision}+\text{recall}}, \end{array}$$

where TP is truly counted cells and TN is the background region that is not considered for cell detection purposes. FP indicates false detections while FN refers to the number of red blood cells that are missed.

Performance parameters are illustrated in 4-7, where accuracy is the proportion of true results used to quantify the relation between automated and manually counted data. Precision is the proportion of truly counted cells among positive results. The recall is often referred to as sensitivity, that is, the proportion of positives for correctly counted cells, and *F*-measure (F1) is the harmonic mean of precision and recall.

## 3. Results and Discussion

All of 66 images were evaluated with Jaccard index where 93.15% similarity coefficient as the overall average of the samples was achieved. The proposed segmentation was compared with Zach's thresholding which is a popular histogram segmentation algorithm employed for RBC segmentation [18]. The *k*-means clustering algorithm with *k* = 2 was also used for comparison. The proposed study was further compared with a background segmentation of a recent study by Molina et al. The recent study employed Otsu's thresholding with preprocessing and postprocessing steps. In order to compare the algorithm performance on our dataset, the recent study's background segmentation was built along with preprocessing and postprocessing steps. The border cleaning operation, on the other hand, was not included considering ground truth data included RBCs at the border [19].  Table 1 illustrates the background segmentation performance of these algorithms.

**Figure 4:**
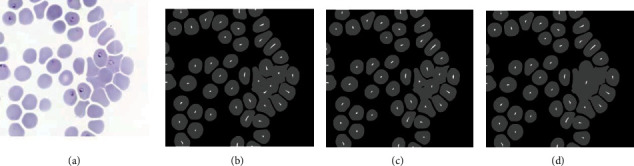
Illustration of extracted foreground markers of red blood cells. The original image (a), extracted markers on the proposed study (b), *k*-means clustering replacement (c), and classic hole filling replacement (d).

**Figure 5:**
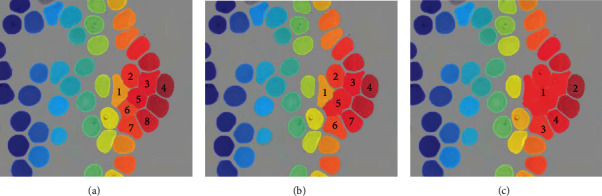
The visual illustration of separated red blood cells by watershed segmentation. The proposed study (a), *k*-means clustering replacement (b), and classic hole filling replacement (c).

The proposed algorithm consists of several steps that complement each other to have successful segmentation of overlapped red blood cells. Our strategy to evaluate the contribution of selective hole filling is to replace it with classic hole filling while keeping the rest of the algorithm steps the same. On the other hand, we also evaluated the contribution of local minimum histogram segmentation by replacing it with *k*-means clustering with *k* = 2. For this comparison, another image set with 15 images with an abundance of overlapping red blood cells was used. The results of this comparison are illustrated in Table 2. The extraction of markers which is associated with the segmentation of overlapping red blood cells and segmentation of overlapping red blood cells are illustrated in Figures 4 and 5, respectively, for visual comparison.

**Figure 6:**
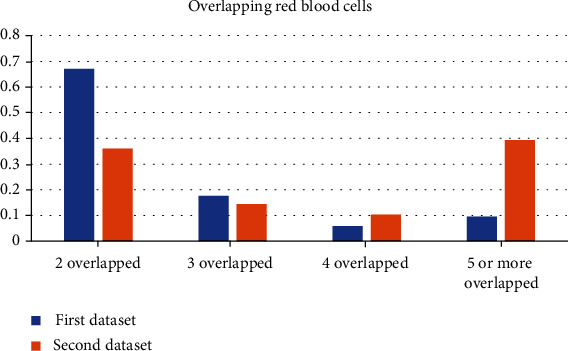
The ratio of different numbers of overlapping RBCs to total number of overlapping RBCs.

For the first dataset, the overlapping ratio of red blood cells versus entire red blood cells was analyzed as 17%, while for the second, 34%. The first dataset, where background segmentation was evaluated consists of 66 images where the second dataset that was used for evaluation of segmentation of overlapped red blood cells consists of 15 images. In Figure 6, the ratios of the different numbers of overlapping RBCs in these two datasets are illustrated. The analysis of 2 datasets shows that the first dataset has an abundance of two overlapped red blood cells while the second one has 5 or more overlapped RBCs.

In this study, novel local minimum histogram segmentation was introduced for background segmentation. We have shown that the selective hole filling approach improved the segmentation of overlapping red blood cells by preserving corner details on red blood cells in clusters. The drawback of this approach is that some RBCs in clusters might have central pallor which can cause oversegmentation issues. Therefore, Hough transform for circle detection was employed to overcome this issue. Considering not all central pallors are circular, this issue remained for some of the RBCs. And a convex hull algorithm was utilized to segment physically disturbed red blood cells in an intact way, which contributed to the quantification of RBCs.

The foreground markers are an important part of watershed segmentation which directly influences the performance of the algorithm. The classic hole filling approach makes extraction of foreground markers difficult that causes undersegmentation issues. The selective hole filling approach was proposed in that regard. The contribution of this approach is illustrated in Figure 4. However, an area estimation of triple overlapping red blood cells is required for the selective hole filling method. Hence, the area of triple overlapped red blood cells should be evaluated for any other dataset.

## 4. Conclusions

The proposed study introduces a novel background segmentation algorithm that outperformed popular background segmentation algorithms that have been utilized for RBC segmentation. Another contribution is the selective hole filling approach, where morphological hole filling was limited to 1-3 RBC-sized objects to preserve inner corners for a high number of overlapped red blood cells. All these improvements were aimed at improving segmentation of overlapping red blood cells by watershed segmentation, which is associated with background and foreground markers. Local minimum histogram segmentation contributes to the background, and selective hole filling contributes to foreground markers. As a result, the segmentation of overlapped red blood cells by marker-controlled watershed segmentation was improved.

## Figures and Tables

**Table 1:** Jaccard similarity coefficient results.

Study	Jaccard similarity coefficient
Proposed study	93.15%
Zack's thresholding	85.53%
*k*-means clustering	88.34%
Molina et al.	87.79%

**Table 2:** Performance evaluation on high number of overlapping cells samples.

Method	TP	FP	FN	Accuracy	Precision	Recall	F1
Proposed study	793	2	7	**0.989**	**0.997**	0.**991**	**0.994**
Classic hole filling replacement	728	2	28	0.960	**0.997**	0.962	0.979
*k*-means clustering replacement	733	15	64	0.902	0.979	0.919	0.948

TP: true positive; FP: false positive; FN: false negative.

## Data Availability

The data used to support the findings of this study are available from the corresponding author upon request.
